# Neuroprotective Effect of Scutellarin on Ischemic Cerebral Injury by Down-Regulating the Expression of Angiotensin-Converting Enzyme and AT1 Receptor

**DOI:** 10.1371/journal.pone.0146197

**Published:** 2016-01-05

**Authors:** Wenjuan Wang, Xiaotang Ma, Jichun Han, Mingjie Zhou, Huanhuan Ren, Qunwen Pan, Chunli Zheng, Qiusheng Zheng

**Affiliations:** 1 Pharmacy School, Shihezi University, Shihezi, China; 2 Department of Pharmacy, the First Division Hospital of Xinjiang Production and Construction Corps, Aksu, Xinjiang, China; 3 College of Life Sciences, Northwest A&F University, Yangling, Shanxi, China; 4 Binzhou Medical University, Yantai, China; 5 Institute of Neurological Disease, Zhanjiang Medical College, Zhanjiang, Guangdong, China; 6 Weifang Medical University, Weifang, China; Indian Institute of Integrative Medicine, INDIA

## Abstract

**Background and Purpose:**

Previous studies have demonstrated that angiotensin-converting enzyme (ACE) is involved in brain ischemic injury. In the present study, we investigated whether Scutellarin (Scu) exerts neuroprotective effects by down-regulating the Expression of Angiotensin-Converting Enzyme and AT1 receptor in a rat model of permanent focal cerebral ischemia.

**Methods:**

Adult Sprague–Dawley rats were administrated with different dosages of Scu by oral gavage for 7 days and underwent permanent middle cerebral artery occlusion (pMCAO). Blood pressure was measured 7 days after Scu administration and 24 h after pMCAO surgery by using a noninvasive tail cuff method. Cerebral blood flow (CBF) was determined by Laser Doppler perfusion monitor and the neuronal dysfunction was evaluated by analysis of neurological deficits before being sacrificed at 24 h after pMCAO. Histopathological change, cell apoptosis and infarct area were respectively determined by hematoxylin–eosin staining, terminal deoxynucleotidyl transfer-mediated dUTP nick end labeling (TUNEL) analysis and 2,3,5-triphenyltetrazolium chloride staining. Tissue angiotensin II (Ang II) and ACE activity were detected by enzyme-linked immunosorbent assays. The expression levels of ACE, Ang II type 1 receptor (AT1R), tumor necrosis factor-α (TNF-α), interleukin-6 (IL-6), and interleukin-1β (IL-1β) were measured by Western blot and real-time PCR. ACE inhibitory activity of Scu in vitro was detected by the photometric determination.

**Results:**

Scu treatment dose-dependently decreased neurological deficit score, infarct area, cell apoptosis and morphological changes induced by pMCAO, which were associated with reductions of ACE and AT1R expression and the levels of Ang II, TNF-α, IL-6, and IL-1β in ischemic brains. Scu has a potent ACE inhibiting activity.

**Conclusion:**

Scu protects brain from acute ischemic injury probably through its inhibitory effect on the ACE/Ang II/AT1 axis, CBF preservation and proinflammation inhibition.

## Introduction

Traditional Chinese medicine (TCM), which develops related theories from long-term clinical practices, has healed people from variety of diseases. Today, TCM still serves as a fruitful therapy resource for multigene disease, such as cardiovascular disease [1] and cancer [2]. Herbal therapy with conventional small molecules has been shown to reduce side effects and/or to improve pharmacological effects. However, the relatively limited knowledge of TCM pharmacology has restricted its wide application in clinical practice. Therefore, emphasizing the mechanistic study of TCM on the molecular level is desired for the discovery of new drugs.

Stroke, sometimes referred to as cerebrovascular accident, cerebrovascular insult, or colloquially brain attack, is the loss of brain function because of a disturbance in the blood supply to the brain. Nowadays, stroke is the second leading cause of death and disability worldwide [3,4]. It threatens human health and quality of life, and 87% of stroke-related deaths occur in low-income and middle-income countries [5]. Hence, search for novel approaches for the prevention and treatment of stroke is extremely urgent.

Recent studies have shown that angiotensin-converting enzyme (ACE)of the renin–angiotensin system plays an important role in stroke. ACE, which converts angiotensin I (Ang I) to angiotensin II (Ang II), is closely related to brain edema, inflammation reaction, and neuron apoptosis after ischemic stroke. Ang II has two specific receptors, namely, Ang II type 1 receptor (AT1R) and Ang II type 2 receptor (AT2R). After binding to AT1R, Ang II may cause ischemic injury via inducing local cerebrovascular vasoconstriction and dysfunction [6,7]. Brain AT1 receptor over-activity leads to inflammation [8] and cell apoptosis [9]. The function of AT2R is complex and still requires investigation. In general, AT2R is presumed to cause vasodilatation and decrease apoptotic rate action, as well as exert neuroprotective effects [10]. Inhibition of ACE also offers neuroprotection by reducing the formation of Ang II. Inhibition of active ACE might reduce risk in humans and reduce the associated disability after stroke [11–15]. Recent clinical studies have revealed that ACEI ameliorates acute ischemic injury [16], partially partially restores age-related alterations in cerebrovascular regulation [17], reduces inflammation [18], and prevents blood–brain barrier breakdown [19].

The flavonoid Scutellarin (Scu) is a major active ingredient extracted from the Chinese herb *Erigeron breviscapus* (Vant.) Hand Mazz. Scu has been used for the clinical treatment of cerebrovascular and cardiovascular diseases, such as stroke. This herb and Scu exert protective effects on brain ischemia or ischemia/reperfusion [20–22]. However, the specific ingredients of a particular herb, as well as the way by which this herb used in disease treatment, remain unknown in most cases. Moreover, the proteins that determine the function of the herbal drug are unclear. Thus, a computer target predictive approach is very useful in identifying drug targets.

This study aimed to examine whether Scu protects against ischemic brain injury explore the underlying molecular mechanisms on a permanent middle cerebral artery occlusion (pMCAO) rat model.

## Materials and Methods

### Animals

All experimental procedures were performed in accordance with the Guidelines for the Care and Use of Laboratory Animals of Shihezi University. The protocol was approved by the Committee on the Ethics of Animal Experiments of the Shihezi University. All surgery was performed under 10% chloral hydrate anesthesia, and all efforts were made to minimize suffering.

Adult Sprague–Dawley rats weighing 230–280 g were obtained from Xinjiang Medicine University Medical Laboratory Animal Center (License Number: SCXK (xin) 2011–0003). The animals were housed in a room with 22°C–25°C temperature, 50%–60% relative humidity, and 12 h/12 h light/ dark cycle. The rats had free access to food and water.

### Drug targeting

To accurately predict the target profiles of Scu, an integrated drug targeting procedure based on our *in silico* ligand-target chemogenomic (LTC) prediction model and public database interrogation strategy was developed by using the following steps. (1) The LTC model was constructed based on two powerful methods, namely, Random Forest (RF) and support vector machine (SVM), by integrating the chemical, genomic, and pharmacological information, which could efficiently predict drug targets on a large scale [23]. Proteins with an output value of SVM > 0.7 or RF > 0.8 were listed as potential targets. (2) STITCH 4 (Search Tool for Interacting Chemicals), a resource that explores the known and predicts interactions of chemicals and proteins derived from experiments, databases, and literature, was used.

**Fig 1:**
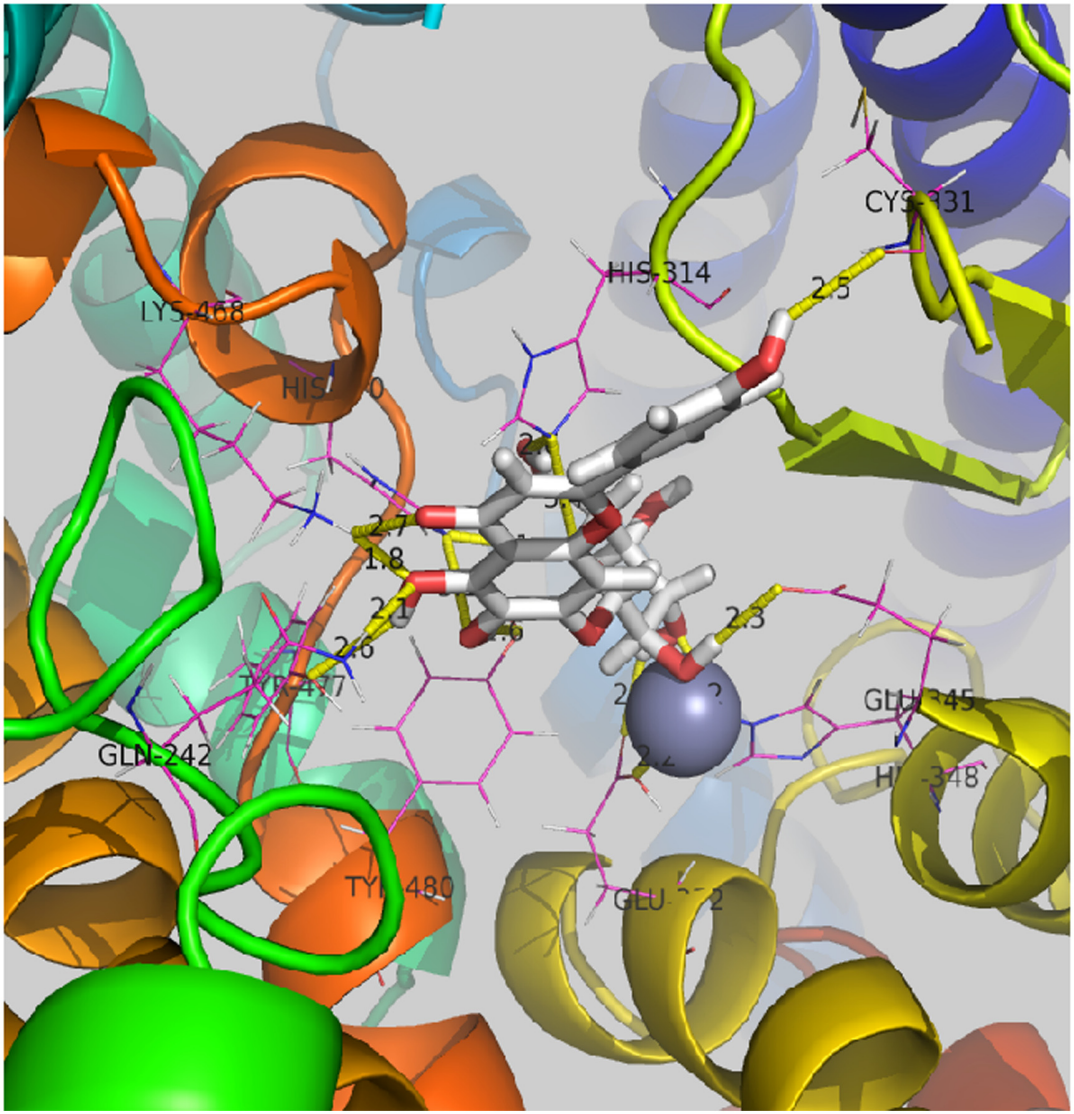
Molecular mode of Scu in the binding site of ACE. The dashed lines show the formation and distance of hydrogen bonds. Active site amino acid residues represent lines.

The obtained target proteins were validated by docking simulation through AutoDock software (version 4.2, http://autodock.scripps.edu/). The protein structures were directly extracted from the RCSB protein data bank (http://www.rcsb.org/pdb/home/home.do), with their resolutions carefully checked. The area of docking was defined as a 60 × 60 × 60 3D grid centered around the ligand binding site with a 0.375 Å grid space. All bond rotations for the ligands were ignored, and the Lamarckian genetic algorithm (LGA) was employed for each simulation [24]. Scu was predicted to target ACE, as supported by literature research. Docking simulation also showed that Scu selectivity binds to ACE with high affinity. These results indicate that ACE is the specific target of Scu (Fig 1).

### Chemicals and Reagents

Scu (98% pure) was purchased from CHENGDU MUST BIO-TECHNOL CO., LTD. (Chengdu, China). Scu was dissolved in saline before use. 2,3,5-Triphenyltetrazolium chloride (TTC) was bought from Sigma Chemical Co. (St. Louis, MO). Antibodies against ACE, AT1R, TNF-α, IL-1β, IL-6, and β-actin were obtained from Cell Signaling Technology (1:10000, Beverly, MA, USA). All other chemicals and reagents were of analytical grade.

### Drug treatment and surgical operation

The animals were randomly divided into five groups (12 rats for each group) for oral gavage administration of Scu: sham, model, model + Scu (25 mg/kg), model + Scu (50 mg/kg), and model + Scu (100 mg/kg). The sham and model groups received normal saline i.g. at the same volumes and time points as the Scu groups. Drug or normal saline was administered once a day for 7 d. The operation was performed 1 h after the last Scu administration.

pMCAO was induced by using the intraluminal filament technique described by Longa [25] with slight modifications. The rats were anesthetized with 10% chloral hydrate (350 mg/kg, i.p.). Although there is a lack of controlled studies relative to the anesthetic quality of chloral hydrate, it is a hypnotic agent that has been used in physiological experiments in laboratory animals, particularly in rodents. A recent report showed that isoflurane exerted a more pronounced analgesic effect than chloral hydrate, but no difference was observed in 24 h survival rate, success of ischemia, or infarct volume reduction between both anesthetics in the middle cerebral artery occlusion model [26]. After a ventral midline neck incision, the right common carotid artery (CCA), the external carotid artery and the internal carotid artery (ICA) were carefully exposed and dissected away from adjacent nerves. Microvascular aneurysm clips were applied to the right CCA and the ICA. A filament rounded by gentle heating near a flame was introduced through a small incision into the CCA and then into the ICA. The suture was inserted 18–20 mm from the carotid bifurcation, which was confirmed by mild resistance to occlude the MCA. The sham-operated rats underwent the same procedure, except that the filament was inserted. During the surgery, body temperature was regulated at 37°C by a heating pad and a warm light. The rats were allowed free access to food and water after the incision was closed.

### Measurement of Neurological Deficits

An observer who was unaware of the experimental treatment groups assessed the neurological deficits after 24 h of ischemia. Neurological findings were scored on a five-point scale [25]: no neurological deficit = 0, failure to extend right paw fully = 1, circling to right = 2, falling to right = 3, did not walk spontaneously and had depressed levels of consciousness = 4.

### Determination of infarct areas

The rats were euthanized 24 h after pMCAO. The brains were quickly removed and sliced into six 2 mm-thick coronal sections. The sections were incubated in 1% TTC at 37°C for 30 min. The stained brain sections were fixed with 10% formalin solution. Normal brain tissue was dyed bright red, and the unstained area was defined as the ischemic lesion. Infarct areas were measured using Photoshop image analysis software. The infarct areas were calculated from the entire area (%).

### Cerebral blood flow measurement

Lasser Doppler perfusion monitor (PeriCam PSI System) was used to measure cerebral blood flow (CBF) as previously described [27,28]. Briefly, 24 h after pMCAO surgery, a crossing skin incision was made on the head of rat to expose the whole skull under anesthesia. Laser scanning imaging measurements were performed on the intact skull. The scan range was 2.0 cm ×1.4 cm. The time required to measure the blood flow of the certain area and generate the image was 1 min. Within the time, the mean blood perfusion of the ischemic hemisphere was calculated by the PeriCam PSI system (Perimed, Sweden).

### Blood pressure detection

Arterial systolic blood pressure (SBP) was measured by a noninvasive tail artery blood pressure measurement system (ZH-HX-Z, Huaibeizhenghua, China) according to the manufactory protocol [29,30] Briefly, rats were kept in a warm environment (40°C) for 15–20 min before the measurement of SBP. Constant pressure was applied to the blood pressure system connected to an occlusion cuff and a biological information collection and processing system (MD3000, Huaibeizhenghua, China). The pressure at the first recorded emerging pulse was defined as SBP. The SBP of each rat was measured at three time points: 1 h before Scu administration, 7 days after Scu administration and 24 h after pMCAO surgery.

### Histological assessment

After 24 h of ischemia, the rats were overdosed with anesthetic and perfused with 4% paraformaldehyde in 0.1 M phosphate buffer solution (PBS, pH 7.4). Brains were removed, fixed in 4% paraformaldehyde at 4°C for 24 h, and cut into equally spaced blocks. Paraffin-embedded blocks were cut into a series of 5 μm-thick slices, stained with HE, and observed under a 200× light microscope.

### Terminal deoxynucleotidyl transfer-mediated dUTP nick end labeling (TUNEL) analysis

TUNEL staining was performed 24 h after ischemia by using In Situ Cell Death Detection Kit (Roche, Mannheim, Germany) in accordance with the manufacturer’s protocol. As previously described [31], the sections were treated with 10 mmol/L protease K for 15 min after deparaffinization and rehydration. The slices were incubated with TUNEL reaction mixture for 1 h at 37°C. The sections were washed thrice in PBS for 10 min each, and color development was performed in the dark with 3,39-diaminobenzidine. Hematoxylin was used for counter-staining, after which the slices were finally mounted onto gelatin-coated slides and dried in the dark. TUNEL-positive apoptotic cells exhibited brown nuclear or cytoplasmic staining. The sections were photographed using an optical microscope. We counted all TUNEL-positive cells in five different fields of each target area (the cerebral cortex of the right hemisphere). TUNEL-positive cells were expressed as percentage of total cell count (400×).

### Assay for ACE activity and ANG II level

ACE activity and ANG II level in ischemic hemisphere tissue were determined using assay kits (Tsz Biosciences, Greater Boston, USA) in accordance with the manufacturer’s instructions.

### Real-time PCR analysis

Total RNA of ischemic brain was extracted using Trizol reagent (Sangon Co., Shanghai, China). RNA quality was tested using the A260/A280 ratio and 1.5% agarose gel electrophoresis, and cDNA synthesis was performed using Moloney murine leukemia virus reverse transcriptase with a First-Strand cDNA Synthesis Kit (Sangon Co., Shanghai, China) in accordance with the manufacturer’s protocol. Relative gene expression was quantified using real-time PCR (Rotor Gene Q, QIAGEN, USA) with a QuantiFast SYBR-Green PCR Kit (QIAGEN), thereby enabling real-time detection of PCR products according to the manufacturer’s instructions. The relative amount of target mRNA was measured by the ΔΔCT method and normalized to GAPDH mRNA levels. The PCR primers (synthesized by Shanghai Sangon Biotechnology Co., Ltd.) were as follows: GAPDH (sense 5’-AGA CAG CCG CAT CTT CTT GT-3’, antisense 5’-CTT GCC GTG GGT AGA GTC AT-3’), ACE (sense 5’-TTG ACG TGA GCA ACT TCC AG-3’, antisense 5’-CAG ATC AGG CTC CAG TGA CA-3’), AT1R (sense 5’-TGA GCA CGC TTT CTT ACC-3’, antisense 5’-CTG CTT AGA TCC TGA GGC-3’), TNF-α (sense 5’-GCG TGT TCA TCC GTT CTC TA-3’, antisense 5’-CGT CTC GTG TGT TTC TGA GC-3’), IL-6 (sense 5’-GTC AAC TCC ATC TGC CCT TC-3’, antisense 5’-GTG GGT GGT ATC CTC TGT GA-3’), and IL-1β (sense 5’-TGT CCT GTG TGA TGA AAG ACG-3’, antisense 5’-CTG CTT GAG AGG TGC TGA TG -3’). The data were presented as the fold change of the shams. All experiments were carried out in triplicate.

### Western blot analysis

The protein was obtained from the ischemic hemisphere to analyze the levels of ACE, AT1R, TNF-α, IL-1β, and IL-6. Tissue was homogenized in lysis buffer (50 mM Tris-HCl, pH 7.6, 0.5% Triton X-100, and 20% glycerol) and then centrifuged (15,000 g, 15 min, 4°C). The supernate containing whole-cell protein extracts was extracted and boiled for 15 min to denature the protein. Total protein concentration was determined using the Bradford method. Protein samples were separated using 12% SDS–polyacrylamide gel electrophoresis and then electrotransferred onto nylon membranes. The membranes were blocked with 5% skimmed milk blocking buffer at room temperature for 1 h and then incubated with primary antibodies overnight (18 h) at 4°C. After washing with TBST buffer, the corresponding secondary antibodies were used to identify primary antibody binding. The blots were visualized with ECL-plus reagent. We used β-actin to demonstrate equal protein loading. Results were expressed as relative density to β-actin.

### Determination of ACE inhibitory activity of Scu in vitro

ACE inhibitory activity of Scu was was performed in a 96-well microplate. A mixture consisting of 40 μL of ACE 0.1U/mL in 80 mM HEPES (PH 8.3) buffer and 160 μL of Scu (100 μM, 50 μM, 25 μM, 12.5 μM and 6.25 μM) in 80 mM HEPES (PH 8.3) buffer was preincubated for 5 min at 37 ℃. A blank sample was prepared by replacing the inhibitor solution with the 80 mM HEPES (PH 8.3) buffer. For FAPGG hydrolysis determination, a 50 μL aliquot of the 200 μL mixture was added to 50 μL of FAPGG 1 mM in 80 mM HEPES (PH 8.3) buffer. The micro plate was shaken 20 s, hydrolysis of FAPGG by ACE was quantified by recording the decrease of absorbance at 340 nm within 30 min.

$$\begin{array}{l} \text{ACE inhibiting activity (\%)}=\frac{\Delta B-\Delta S}{\Delta B}\times 100 \end{array}$$

The ΔS and ΔB are represents the ACE activity change rate of samples group and blank group. The IC50 value was defined as the concentration of inhibitor required to reduce the slope by 50% in the well.

### Statistical Analysis

Data were expressed as mean ± S.E. Statistical comparisons were performed with one-way ANOVA, followed by Student–Newman–Keuls method or post-hoc test. *P* < 0.05 was considered statistically significant.

## Results

### Scu selectivity binds to ACE with high affinity

The obtained target proteins were validated by docking simulation through AutoDock software (version 4.2, http://autodock.scripps.edu/). The protein structures were directly extracted from the RCSB protein data bank (http://www.rcsb.org/pdb/home/home.do), and their resolutions were carefully checked. The area of docking was defined as a 60 × 60 × 60 3D grid centered around the ligand binding site with a 0.375 Å grid space. All bond rotations for the ligands were ignored, and the LGA was employed for each simulation [24]. Scu was predicted to target ACE, as supported by literature. Docking simulation also showed that Scu selectively binds to ACE with high affinity. These results indicate that ACE is the specific target of Scu (Fig 1).

### Effects of Scu on cerebral infarct area and neurological scores

**Fig 2:**
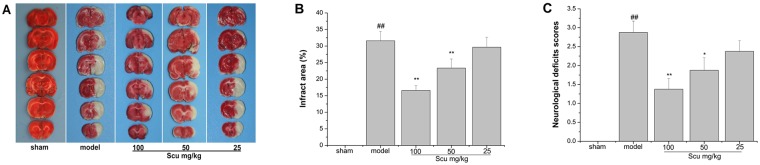
Effects of Scu on permanent focal cerebral ischemia in rats. (A) Representative coronal brain sections stained with TTC after 24 h of pMCAO. Red-colored region in the TTC-stained sections indicates no ischemic portion of brain, and pale-colored region indicates ischemic portion. (B) Evaluation of infarct area 24 h after pMCAO. Infarct areas were measured using Photoshop image analysis software. (C) Neuroprotective effects of Scu on neurological deficit scores in pMCAO rats. Data are expressed as means ±S.D, ^##^P < 0.01 vs. sham group, *P < 0.05, **P < 0.01 vs. model group, n = 8.

The cerebral infarct area was measured 24 h after pMCAO by TTC staining (Fig 2A). No infarct area was observed in the sham group, thereby indicating that the operation did not influence infarct size. Scu at 100 and 50 mg/kg prominently decreased the cerebral infarct area (P < 0.01 or 0.05 vs. model group, Fig 2B). As shown in Fig 2C, the neurological deficit score of the model group significantly increased 24 h after pMCAO (P < 0.01 vs. sham). The neurological deficit scores significantly improved in the Scu-treated groups (100 or 50 mg/kg) (P < 0.01 or 0.05 vs. model group).

### Effects of Scu on cerebral blood flow and arterial blood pressure

**Fig 3:**
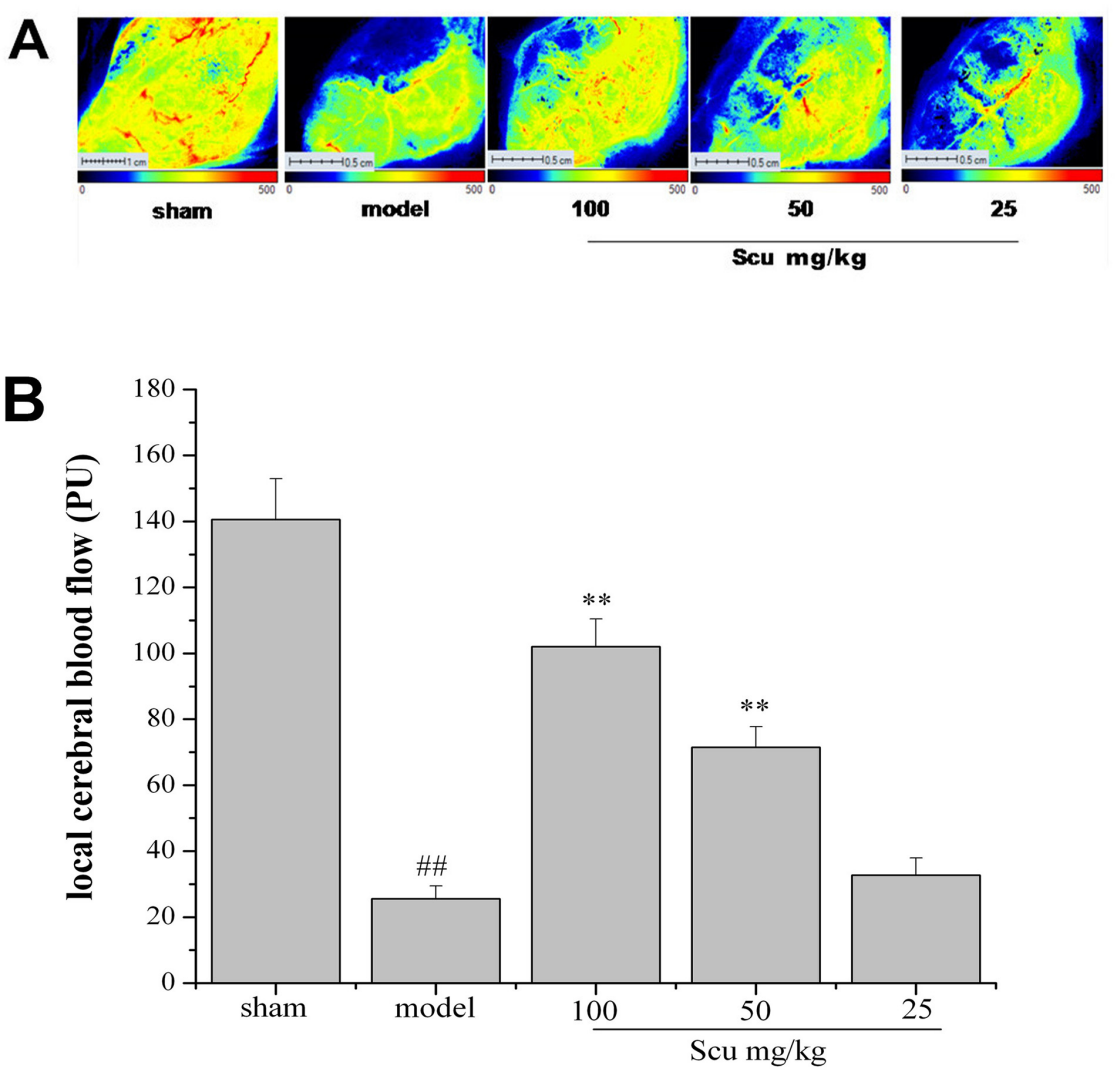
Effects of Scu on cerebral blood flow. (A) Laser scanning images of CBF in whole cortex 24 h after operation in sham-operated rat, pMCAO rat and pMCAO rat administrated with 100, 50 and 25mg/kg Scu. CBF recovery can be seen in the pMCAO rat received 100, 50 mg/kg Scu. (B) Ischemic hemisphere showed significant higher blood flow in pMCAO rat received 100, 50 mg/kg Scu when compared with model group. Data are expressed as mean ± S.D. ^##^P < 0.01 vs. sham, **P<0.01 vs. model.

The cerebral blood flow (CBF) was measured 24 h after pMCAO by Lasser Doppler perfusion monitor (Fig 3). As shown in Fig 3, the CBF of the model group significantly decreased 24 h after pMCAO. Scu at 100 and 50 mg/kg prominently improved the ischemic hemisphere blood flow compared with the model group (P < 0.01 or 0.05 vs. model, Fig 3).

**Fig 4:**
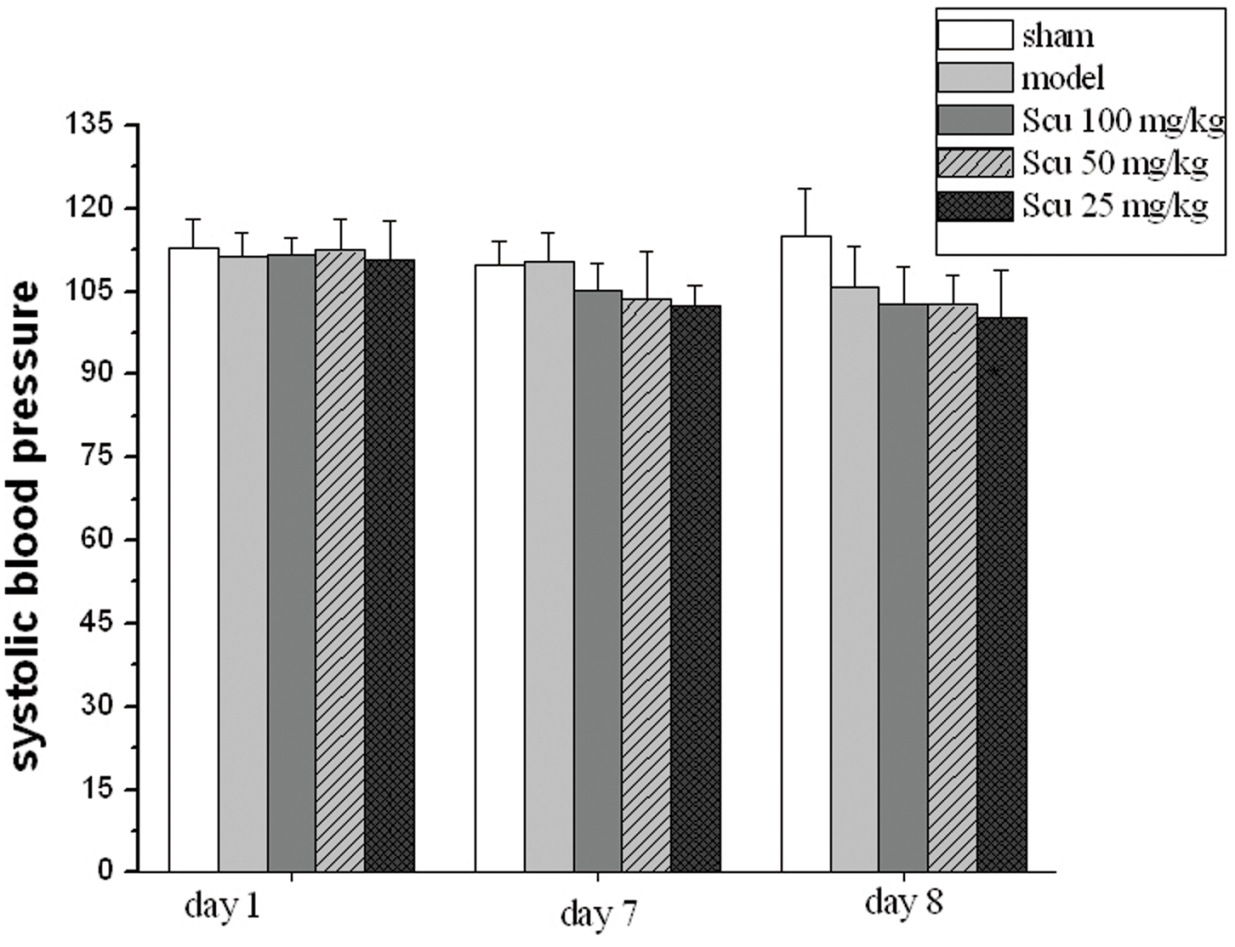
Effects of Scu on blood pressure in pMCAO rat. The SBP was measured by noninvasive blood pressure system at three time points in pMCAO rat: 1 h before administrated on day 1, seven days after administered and 24 h after pMCAO surgery. There was no significant difference between pMCAO rat with Scu administrated and without Scu treatment.

The arterial blood pressure was measured by the noninvasive tail cuff method. The results showed that the pMCAO surgery or Scu has no significant influence on the SBP of the rats (Fig 4).

### Effects of Scu on histopathological changes and neuronal apoptosis

**Fig 5:**
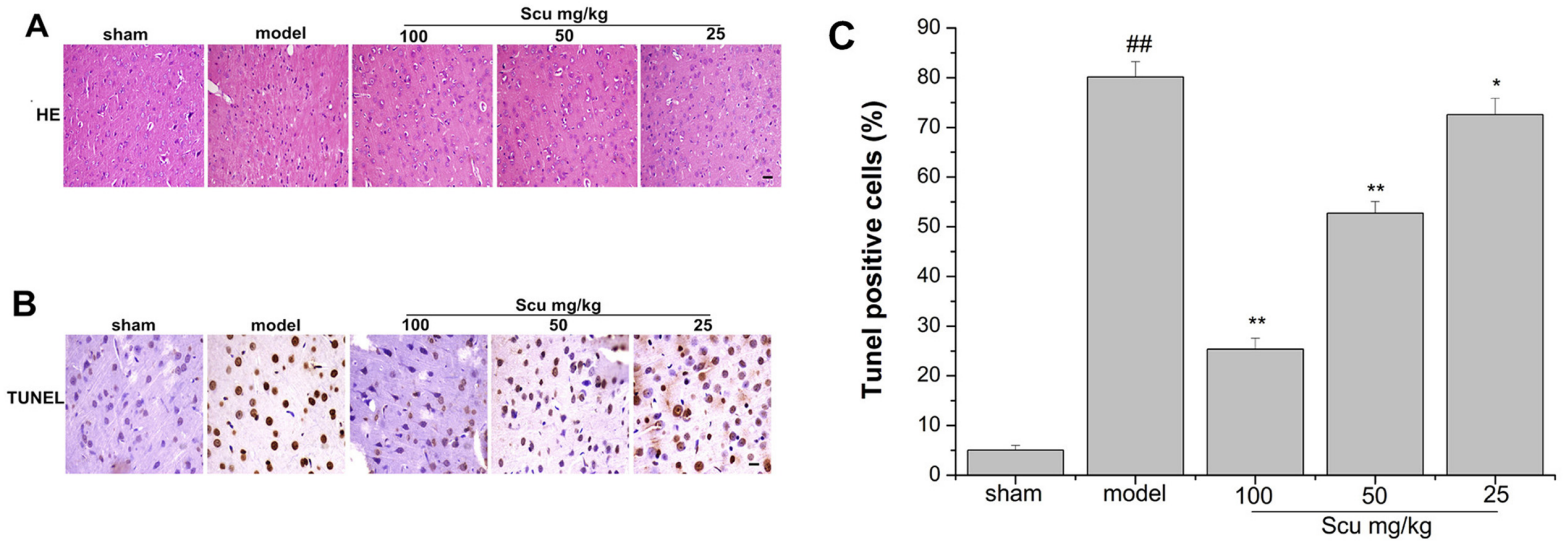
Effects of Scu on histopathological changes in pMCAO rats. (A) Representative coronal sections stained with HE after 24 h of pMCAO (×200 magnifications, scale bars = 62.4 μm). (B) Representative images of TUNEL staining under microscope (×400 magnifications, scale bars = 31.2 μm) and quantitative immunoreactivities of TUNEL-positive cells (C) after 24 h of pMCAO. Apoptotic cells exhibited brown nuclear or cytoplasmic staining. Data are expressed as means ±S.D., ^##^P < 0.01 vs. sham group, *P < 0.05, **P < 0.01 vs. model group.

The histopathology of the cortex was examined by HE staining (Fig 5A). No histopathological abnormalities were observed in the sham group. The model group mostly showed shrunken neurons, dissolved cytoplasm, and pycnotic nucleus with triangular or irregular shape. Scu treatment markedly reduced these pathomorphological changes.

As shown in Fig 5B, a few apoptotic neurons existed in sham group, whereas the number of apoptotic cells in the model group increased significantly compared with that in the sham group. The number of apoptotic neurons decreased significantly in Scu treatment group (Fig 5C, p < 0.01 vs. groups treated with Scu at 100 and 50 mg/kg; p < 0.05 vs. group treated with Scu at 25 mg/kg). These results suggest that Scu can dose-dependently reduce apoptotic cell death induced by pMCAO.

### Scu treatment dose-dependently inhibits the elevation of ACE activity induced by pMCAO

The mean ACE activity levels in the sham, model, and in the groups treated with Scu at 100, 50, and 25 mg/kg were 25.71 ± 3.64, 77.86 ± 5.68, 35.91 ± 2.28, 45.79 ± 4.23, and 65.82 ± 5.48 u/L, respectively. The ACE activity increased significantly in the model group compared with the sham group (p < 0.01), whereas the activity of ACE decreased significantly in Scu-treated groups (p < 0.01 vs. groups treated with Scu at 100 and 50 mg/kg; p < 0.05 vs. group treated with Scu at 25 mg/kg).

### Scu treatment dose-dependently decreases Ang II level induced by pMCAO

The Ang II levels in the sham, model, and groups treated with Scu at 100, 50, and 25 mg/kg were 515.96 ± 18.37, 785.90 ± 55.43, 572.95 ± 21.37, 665.22 ± 30.12, and 717.81 ± 27.77 ng/mL, respectively. The mean ANG II level was higher in the model group in the sham group (p < 0.01). Compared with the model group, the Scu-treated groups had significantly reduced Ang II levels (p < 0.01 vs. groups treated with Scu at 100 and 50 mg/kg; p < 0.05 vs. group treated with Scu at 25 mg/kg) induced by pMCAO.

### Scu dose-dependently reduces the mRNA expression of ACE, AT1R, TNF- α, IL-6, and IL-1β

**Fig 6:**
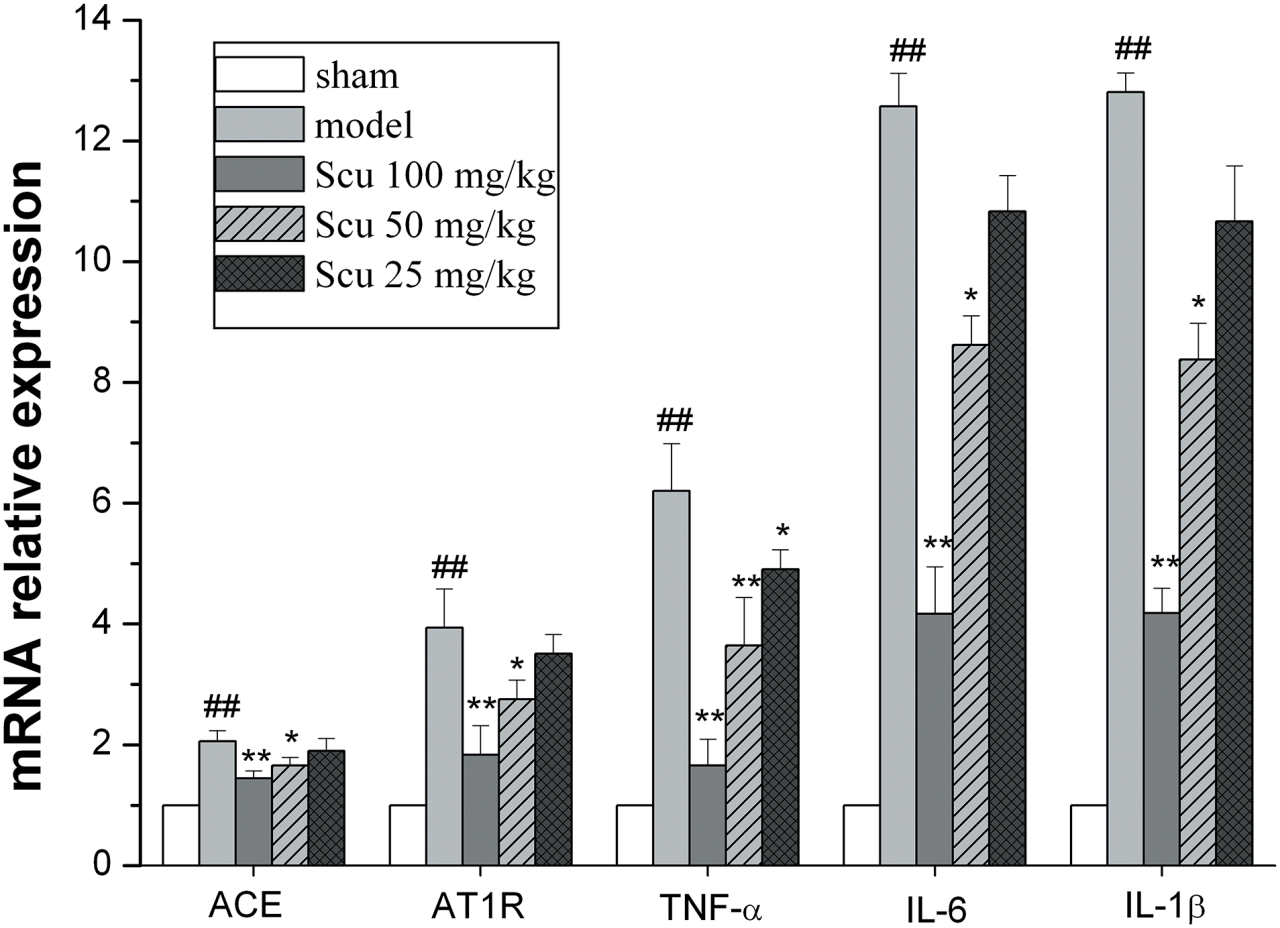
Effects of Scu on mRNA expressions of ACE, AT1R, TNF- α, IL-6, and IL-1β after 24 h of pMCAO. Graphical representation of fold change of ACE mRNA. Each bar represents means ±S. D., ^##^P < 0.01 vs. sham group, *P < 0.05, **P < 0.01 vs. model group.

As shown in Fig 6, the mRNA expression levels of ACE and AT1R, and levels of proinflammatory cytokines TNF-α, IL-1β, and IL-6 were notably higher in the model group than in the sham group 24 h after pMCAO. Scu treatment significantly attenuated these changes.

### Scu dose-dependently reduces the protein expression of ACE, AT1R, TNF-α, IL-1β, and IL-6 in ischemic cerebral tissue

**Fig 7:**
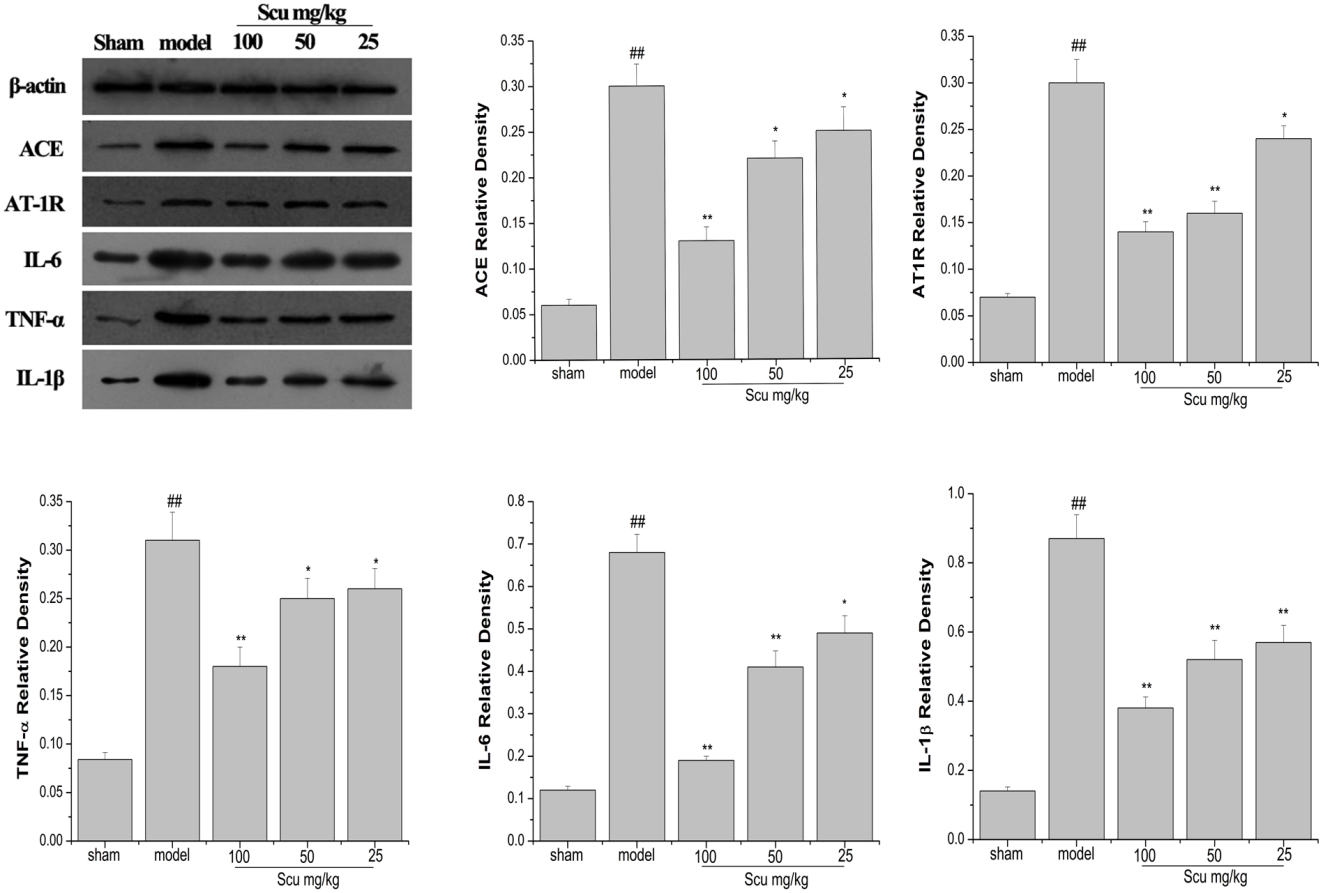
Representative Western blots and quantitative analysis of ACE, AT1R, TNF-α, IL-1β, and IL-6 on the protein level in ischemic brain 24 h after MCAO in rats. Data are expressed as means ±S. D., ##P < 0.01 vs. sham group, *P < 0.05, **P < 0.01 vs. model group.

The protein expression levels of ACE, AT1R, TNF-α, IL-1β, and IL-6 were detected by Western blot analysis. As shown in Fig 7, the protein expression of ACE significantly increased in the model group (p < 0.01 vs. sham group). Treatment with Scu significantly decreased the expression of ACE, AT1R, TNF-α, IL-1β, and IL-6 (Scu at 100 mg/kg vs. model, p < 0.01; Scu at 50 mg/kg vs. model, p < 0.05; Scu at 25 mg/kg vs. model, p < 0.05).

### In vitro ACE inhibitory activities of Scu

As shown in Table 1, the IC50 values of Scu against ACE is 48.13±4.98 μM. This result demonstrates that Scu has a potent ACE inhibiting activity.

**Table 1:** In vitro ACE inhibitory activity (IC_50_) of Scu.

Chemical name	IC50 (μM)
Scutellarin	48.13±4.98

Values are means with their standard deviation.

## Discussion

In this study, we applied AutoDock software to predict ACE as a target of Scu, then further verified in vivo and in vitro. We investigated the effects of Scu on neurological scores, tissue morphology, neuronal apoptosis and infarct area in pMCAO rats. We further explored the effects of Scu on the target of ACE, and its associated downstream inflammatory mediators. The results showed that Scu reduced neurological scores and infarct area, and neuronal apoptosis. We discovered that Scu, which was predicted to target ACE, decreased the gene and protein expression of ACE andAR1R, and the production of ANG II, TNF-α, IL-6 and IL-1β. Moreover, Scu has a potent ACE inhibiting activity in vitro.

Recent research has shown that the ACE is involved in the pathological process of stroke and various cardiovascular diseases, many of which are risk factors for stroke [11,15,32]. ACE is associated with the endothelium and glial cells in the brain [33,34]. ACEI protects brain from cerebral ischemic injury in no-hypertensive [11,14,15], as well as in spontaneously hypertensive rats [35]. We observed that after 24 h of pMCAO, the activity and content of ACE significantly increased in model group, indicated that ACE involved in the ischemic injury. More importantly, we found that Scu down-regulated ACE on both protein and mRNA levels and exerts neuroprotective effects.

The activation of brain Ang II/AT1R may result in cerebral ischemic injury. Cerebral ischemia is a common pathological state revealed as a key factor contributing to stroke [36]. It can cause brain injury, leading to neurology defects, cerebral infarct and neuron death [37]. Our results demonstrated that Scu at doses of 50 and 100 mg/kg significantly reduced infarct volumes and improved neurological deficits in a rat model of cerebral ischemia, indicating the neuroprotective effects of Scu. CBF dysregulation plays key roles in the development of brain damage following cerebral ischemia [38]. Reduction of CBF has been suggested as a predictive marker for stroke progression [39] and is one of the important strategies for limiting ischemic injury [40]. Intriguingly, a dose-dependent improvement in CBF was observed after administration of Scu in the pMCAO rat model. This suggests that Scu could help restoration of local blood supply and therefore contribute to the protective effect on ischemic brain. As mentioned above, the ACE/Ang II/AT1R axis is involved in cerebral ischemic injury[41]. Of note, although our results showed that Scu down-regulated the expression of ACE and AT1R, we did not observe significant changes on the SBP after Scu administration. It suggests that Scu is effective and safety strategy to treat ischemic stroke. Mounting evidence shows that excessive inflammation [42] and apoptosis [43] contribute to the damage caused by ischemic stroke. Ang II /AT1R signaling participates in inflammation and oxidative stress during CNS injury [44,45]. Inhibiting AT1R may protect the brain from further inflammatory cascades that lead to cell damage and apoptosis [46]. In agreement with these results, Scu has been shown obvious antioxidant effect in vitro[47] and in vivo, such as effectively inhibit inflammatory responses [21], oxidative tissue damage [48] and neuronal apoptosis [49] through alleviating oxidative stress. Here, we showed the unequivocal anti-inflammatory action and apoptosis inhibition of Scu in the pMCAO model.

Ischemic stroke triggers inflammatory cytokine formation, in turn, promotes inflammatory cascades that increase cellular toxicity and lead to additional neuronal damage. Inflammatory cytokines (TNF-α, IL-1β, and IL-6) are released by astrocytes, microglia, smooth muscle cells, and endothelial cells [50]. Previous studies have reported that Ang II via AT1R activates NF-κB expression, which is the start of transcription of inflammatory cytokines, leukocyte adhesion, and oxidative stress, thereby worsens vascular endothelial cells and brain tissue ischemia-reperfusion injury [51]. In the present study, we found that Scu attenuated pMCAO-induced cerebral injury and down-regulated the protein and mRNA expressions of IL-1β, IL-6, and TNF-α. Histopathological examination also showed that Scu treatment protected neurons after the onset of cerebral ischemia. This result suggests that Scu reduces inflammatory-related damage. We would like to point out that Ang II is the activator of NADPH oxidase and most of the ACE inhibitors show anti-oxidative effect [52,53]. Therefore, anti-oxidative effect of Scu should be investigated in the future.

**Fig 8:**
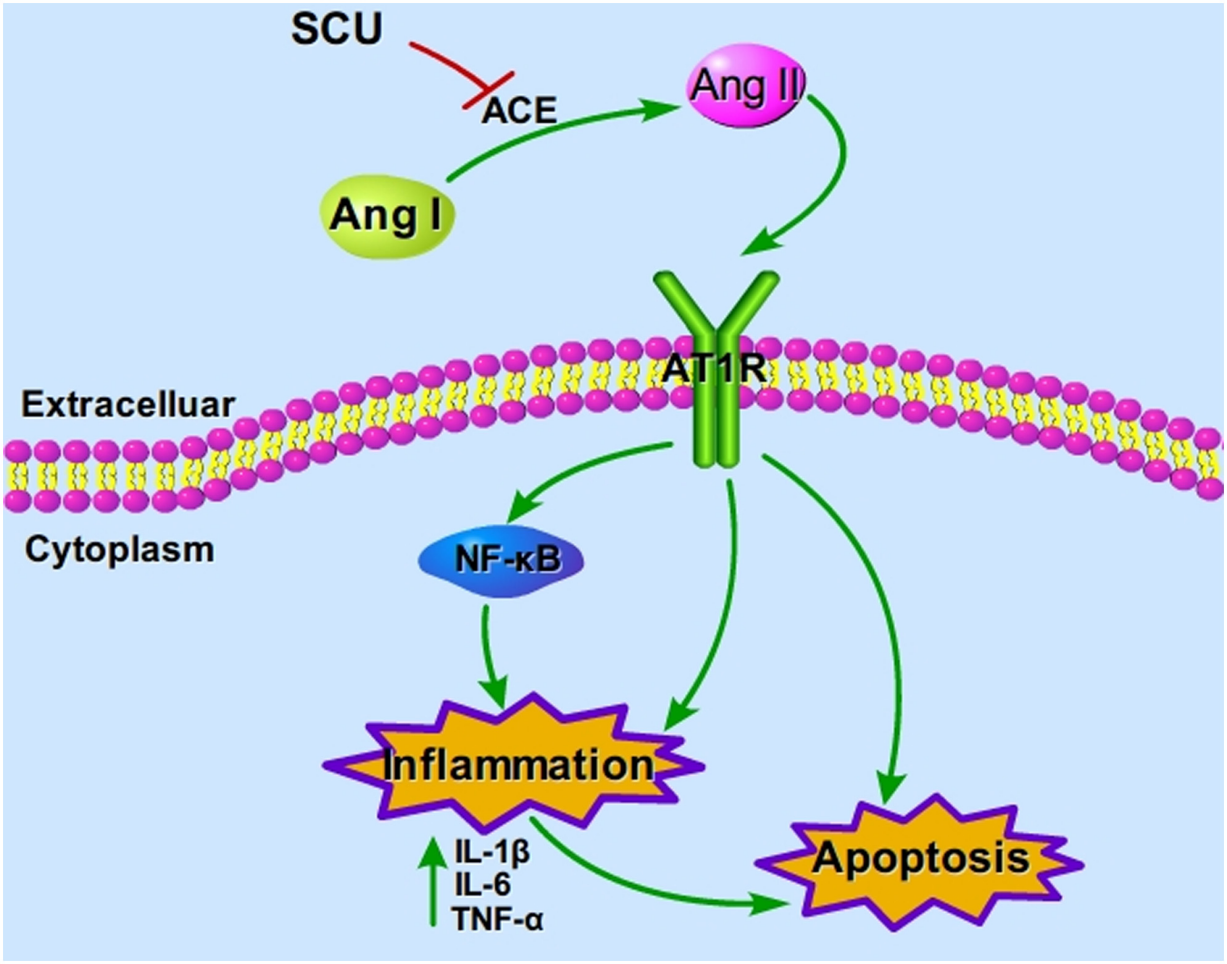
Speculative mechanism of neuroprotective by Scu.

As summarized in Fig 8, Scu protects rat brain from acute ischemic injury by inhibiting the activity of ACE, thereby reducing the formation of Ang II and inhibiting Ang II/AT1R pathway mediated production of proinflammatory factors, such as TNF-α, IL-1β and IL-6.

## Supporting Information

S1 Table Infract area, Neurological deficit scores, CBF and TUNEL positive cells data. (DOC)

S2 Table BP data. (DOC)

S3 Table mRNA expressions of ACE, AT1R, TNF- α, IL-6, and IL-1β data. (DOC)

S4 Table Representative Western blots and quantitative analysis of ACE, AT1R, TNF-α, IL-1β, and IL-6 on the protein level data. (DOC)

S5 Table In vitro ACE inhibitory activities of SCU data. (DOC)
