# Correction: Neuroprotective Effect of Scutellarin on Ischemic Cerebral Injury by Down-Regulating the Expression of Angiotensin-Converting Enzyme and AT1 Receptor

**DOI:** 10.1371/journal.pone.0147780

**Published:** 2016-01-22

**Authors:** Wenjuan Wang, Xiaotang Ma, Jichun Han, Mingjie Zhou, Huanhuan Ren, Qunwen Pan, Chunli Zheng, Qiusheng Zheng

The affiliation for the second and sixth authors is incorrect. Xiaotang Ma and Qunwen Pan are both affiliated with: Institute of Neurological Disease, Guangdong Medical College, Zhanjiang, Guangdong, China.
