# Supplementary material for: Neuroprotective Effect of Scutellarin on Ischemic Cerebral Injury by Down-Regulating the Expression of Angiotensin-Converting Enzyme and AT1 Receptor
Source: PLoS One. 2016 Jan 5;11(1):e0146197. doi: 10.1371/journal.pone.0146197 (PMC4711585; doi:10.1371/journal.pone.0146197)
Supplement: S1 Table — (DOC) [file pone.0146197.s001.doc]

**S1 Table. Infract area, Neurological deficit scores, CBF and TUNEL positive cells data.**

|  | sham | model | Scu 100 mg/kg | Scu 50 mg/kg | Scu 25 mg/kg |
| --- | --- | --- | --- | --- | --- |
| Infract area (%) | 0.0±0.0 | 31.1±5.8 | 16.1±4.1 | 23.1±4.9 | 28.5±4.0 |
| Neurological deficit scores | 0.0±0.0 | 2.9±0.6 | 1.4±0.5 | 1.9±0.3 | 2.3±0.5 |
| CBF(PU) | 140.6±12.4 | 25.6±3.9 | 102±8.4 | 71.5±6.3 | 32.7±5.2 |
| TUNEL positive cells | 5.0±1.0 | 80.1±3.1 | 25.3±2.2 | 52.7±2.3 | 72.5±3.3 |
