# Supplementary material for: Neuroprotective Effect of Scutellarin on Ischemic Cerebral Injury by Down-Regulating the Expression of Angiotensin-Converting Enzyme and AT1 Receptor
Source: PLoS One. 2016 Jan 5;11(1):e0146197. doi: 10.1371/journal.pone.0146197 (PMC4711585; doi:10.1371/journal.pone.0146197)
Supplement: S2 Table — (DOC) [file pone.0146197.s002.doc]

**S2 Table. BP data.**

| groups  blood pressure  (mm Hg) | sham | model | Scu 100 mg/kg | Scu 50 mg/kg | Scu 25 mg/kg |
| --- | --- | --- | --- | --- | --- |
| day 1  (before administered) | 112.8±5.1 | 111.3±4.3 | 111.6±3.1 | 112.4±5.6 | 110.7±7.1 |
| day 7  （before operation） | 109.6±4.4 | 110.2±5.4 | 105.3±4.7 | 103.7±8.5 | 102.4±3.6 |
| day 8  （24h after operation） | 115.1±8.6 | 105.8±7.4 | 102.7±6.8 | 102.6±5.3 | 100.3±8.7 |
