# Supplementary material for: Neuroprotective Effect of Scutellarin on Ischemic Cerebral Injury by Down-Regulating the Expression of Angiotensin-Converting Enzyme and AT1 Receptor
Source: PLoS One. 2016 Jan 5;11(1):e0146197. doi: 10.1371/journal.pone.0146197 (PMC4711585; doi:10.1371/journal.pone.0146197)
Supplement: S3 Table — (DOC) [file pone.0146197.s003.doc]

**S3 Table. mRNA expressions of ACE, AT1R, TNF- α, IL-6, and IL-1β data.**

| groups  fold change | sham | model | Scu 100 mg/kg | Scu 50 mg/kg | Scu 25 mg/kg |
| --- | --- | --- | --- | --- | --- |
| ACE | 1.0±0.0 | 2.1±0.2 | 1.4±0.1 | 1.7±0.1 | 1.9±0.2 |
| AT1R | 1.0±0.0 | 3.9±0.6 | 1.8±0.5 | 2.8±0.3 | 3.5±0.3 |
| TNF- α | 1.0±0.0 | 6.2±0.8 | 1.7±0.4 | 3.6±0.8 | 4.9±0.3 |
| IL-6 | 1.0±0.0 | 12.6±0.5 | 4.2±0.8 | 8.6±0.5 | 10.8±0.6 |
| IL-1β | 1.0±0.0 | 12.8±0.3 | 4.2±0.4 | 8.4±0.6 | 10.7±0.9 |
