# Supplementary material for: Neuroprotective Effect of Scutellarin on Ischemic Cerebral Injury by Down-Regulating the Expression of Angiotensin-Converting Enzyme and AT1 Receptor
Source: PLoS One. 2016 Jan 5;11(1):e0146197. doi: 10.1371/journal.pone.0146197 (PMC4711585; doi:10.1371/journal.pone.0146197)
Supplement: S4 Table — (DOC) [file pone.0146197.s004.doc]

**S4 Table. Representative Western blots and quantitative analysis of ACE, AT1R, TNF-α, IL-1β, and IL-6 on the protein level data.**

| groups  relative density | sham | model | Scu 100 mg/kg | Scu 50 mg/kg | Scu 25 mg/kg |
| --- | --- | --- | --- | --- | --- |
| ACE | 3666.7±175.5 | 20316.4±352.2 | 5574.6±261.4 | 12128.4±280.5 | 12364.6±245.0 |
| AT1R | 2516.5±743.3 | 9356.9±1185.8 | 7880.6±974.4 | 6461.2±1131.3 | 3159.0±958.9 |
| TNF- α | 2136.7±317.5 | 13383.3±646.2 | 4239.2±154.9 | 4888.8±309.6 | 4306.9±763.1 |
| IL-6 | 5889.6±450.2 | 27510.3±474.6 | 11914.5±161.9 | 1789.2±117.3 | 16539.8±180.1 |
| IL-1β | 4281.8±83.4 | 26198.2±197.2 | 7182.0±138.5 | 12014.6±211.3 | 17113.9±361.3 |
