# Supplementary material for: Neuroprotective Effect of Scutellarin on Ischemic Cerebral Injury by Down-Regulating the Expression of Angiotensin-Converting Enzyme and AT1 Receptor
Source: PLoS One. 2016 Jan 5;11(1):e0146197. doi: 10.1371/journal.pone.0146197 (PMC4711585; doi:10.1371/journal.pone.0146197)
Supplement: S5 Table — (DOC) [file pone.0146197.s005.doc]

S5 Table. In vitro ACE inhibitory activities of SCU data.

| IC_50_(μM) | 51.94 | 47.85 | 52.62 | 40.10 |
| --- | --- | --- | --- | --- |
